# Pre- and post-operative gait analysis for evaluation of neck pain in chronic whiplash

**DOI:** 10.1186/1749-7221-4-10

**Published:** 2009-07-17

**Authors:** Ake Nystrom, Glen M Ginsburg, Wayne Stuberg, Stacey Dejong

**Affiliations:** 1 Department of Orthopaedic Surgery and Rehabilitation, University of Nebraska Medical Center, Omaha NE 68198, USA; 2 Division of Plastic and Reconstructive Surgery, University of Nebraska Medical Center, Omaha NE 68198, USA; 3 Munroe-Meyer Motion Analysis Laboratory, University of Nebraska, Lincoln, NE 68588, USA

## Abstract

**Introduction:**

Chronic neck pain after whiplash is notoriously refractory to conservative treatment, and positive radiological findings to explain the symptoms are scarce. The apparent disproportionality between subjective complaints and objective findings is significant for the planning of treatment, impairment ratings, and judicial questions on causation. However, failure to identify a symptom's focal origin with routine imaging studies does not invalidate the symptom per se. It is therefore of a general interest both to develop effective therapeutic strategies in chronic whiplash, and to establish techniques for objectively evaluation of treatment outcomes.

**Methods:**

Twelve patients with chronic neck pain after whiplash underwent pre- and postoperative computerized 3D gait analysis.

**Results:**

Significant improvement was found in all gait parameters, cervical range-of-motion, and self reported pain (VAS).

**Conclusion:**

Chronic neck pain is associated with abnormal cervical spine motion and gait patterns. 3D gait analysis is a useful instrument to assess the outcome of treatment for neck pain.

## Introduction

Serious persistent problems after whiplash trauma to the neck, sometimes referred to as Whiplash Associated Disorders (WAD)[1] is a common and costly condition; estimates indicate an incidence of over 250,000 in the United States, at an annual cost in 2002 of $2.7 billion or close to $10,000 per incident. [2] Although initial symptoms from acceleration-deceleration trauma to the neck may improve spontaneously or with physical therapy over the course of weeks-to-months, [1] chronic and potentially disabling symptoms persist in a significant percentage of all cases. [3,4] A complicating factor, which is also a reason for controversy, is the frequent failure of routine clinical laboratory investigative methods including MRI and electrodiagnostic studies, to objectively identify the cause of pain and other symptoms. [5,6]

Although not a universal finding, stiffness of the neck and shoulders is a common sequela of whiplash. [5-10] Using 3D motion analysis techniques, Dall'Alba et al. [11] identified significant limitations with a particular pattern of cervical range of motion among patients with WAD, but also pointed out that their results do not provide an explanation for the loss of neck mobility. In a study where similar techniques were applied, Gargan et al found that cervical range of motion and psychological scores at three months were predictive of clinical outcomes at 2 years. [11] Their findings were confirmed by Tomlinson et al in a follow-up study on the same cohort, 7.5 years later. [9]

Existing data suggest that neck stiffness in WAD may be an expression of pain inhibition from soft tissue injury and painful muscle spasm without pathology of the spine. Thus, injections of Botox^® ^to trigger points in superficial neck muscles have been shown to provide temporary but significant decrease in pain and increase in cervical ROM,[8] with similar effect of short duration from injections of local anesthetic to myofascial trigger points in the neck. [12] While rarely a definitive solution to problems associated with the chronic whiplash syndrome, such injections may be helpful in identifying focal origin(s) of soft-tissue pain. [12,13]

3D motion analysis represents the diagnostic gold standard for conditions that affect the kinematics of the lower extremities, pelvis and trunk. Using this technology, several investigators have confirmed that deviations from normal gait mechanics also affect the compensatory movements of the head and neck. [14,15] Other studies have demonstrated that temporal and spatial changes in gait are complimented in the neck through input from the vestibulo-ocular reflex (VOR) for stabilization of gaze during angular movements, [16] while head position is controlled by the cervicocollic reflex (CCR), vestibulocollic reflex (VCR) and optocollic reflexes (OCR) through proprioceptive, vestibular and ocular mechanisms. [14,16] Whether variations in gait parameters are voluntary (due to changes in terrain, gait speed, direction, etc.) or represent deviations from "normal" kinematics (changes in temporal distance measures of walking or joint movement from disease, injury, or surgery), they will, through reflex mechanisms, result in adaptive changes in the kinematics of the cervical spine.

The effect of lower segment dysfunction on the upper body kinematics has been previously investigated in normal controls and in patient groups with musculoskeletal disorders. [17-19] We have not, however, found any studies exploring if standard gait parameters are impaired as a result of upper body dysfunction, The present investigation was designed for that purpose and, secondly, to assess the usefulness of computerized 3D gait analysis to objectively monitor outcomes of treatment for neck pain.

## Methods

### Subjects

Participants were recruited among patients referred to University of Nebraska Medical Center for treatment of chronic neck pain after whiplash (WAD II–III, Table 1). Inclusion criteria are summarized in Table 2.

**Table 1:** Classification of Whiplash Associated Disorders (WAD)

0	No complaints. No objective physical signs
**I**	Pain. No objective physical signs.

**II**	Pain. Objective musculoskeletal signs, e.g. stiffness.

**III**	Pain. Objective neurological signs, e.g. weakness, numbness, absent tendon reflexes.

**IV**	Pain. Radiological evidence of skeletal injury or dislocation.

**Table 2:** Inclusion criteria

Age 19 or older
Neck pain precipitated by whiplash trauma

Failure of conservative treatment for more than one year

Absence of gross neurologic signs

Absence of gross radiological (MRI) pathology

The study group consisted of twelve consecutive patients (10 F, 2 M) ages 26 to 67 (mean 44.9 ± 12.8). All subjects were able to understand simple commands and ambulate independently with or without assistive devices.

### Treatment

Areas of intense focal tenderness, generally in the lower cervical paraspinal musculature or horizontal segment(s) of the trapezius muscle(s), were preoperatively mapped through diagnostic injections of local anesthetic (Marcaine^® ^0.25 mg/ml). In a surgical procedure designed to identify and eliminate focal pain generators, the 'tender points' were thereafter addressed during an operation that generally included exploration, neurolysis and decompression of the spinal accessory nerve and/or dorsal sensory branches of cervical nerve roots at their passage through fibrotic trapezius fascia, and trapezius fasciectomy.[13,20] In order to optimize the outcome of treatment, all patients participated actively with the surgeon in the operating room to identify focal areas of pain. No sedation, analgesia or local anesthetic was used during these key portions of the procedure.

### Data collection

Three dimensional motion analyses were carried out using a six camera Vicon system (60 Hz), Vicon Workstation and Polygon software, and the Vicon Plug-In-Gait full body biomechanical model to collect pre- and postoperative data pertaining to gait (speed, cadance and step length), and cervical range-of-motion (degrees from resting position). Pain was assessed with a linear Visual Analogue Scale (VAS) graded 0–1. The evaluations were performed one week before, and 1–10 weeks (27.7 ± 21.6 days) after surgery.

**Figure 1:**
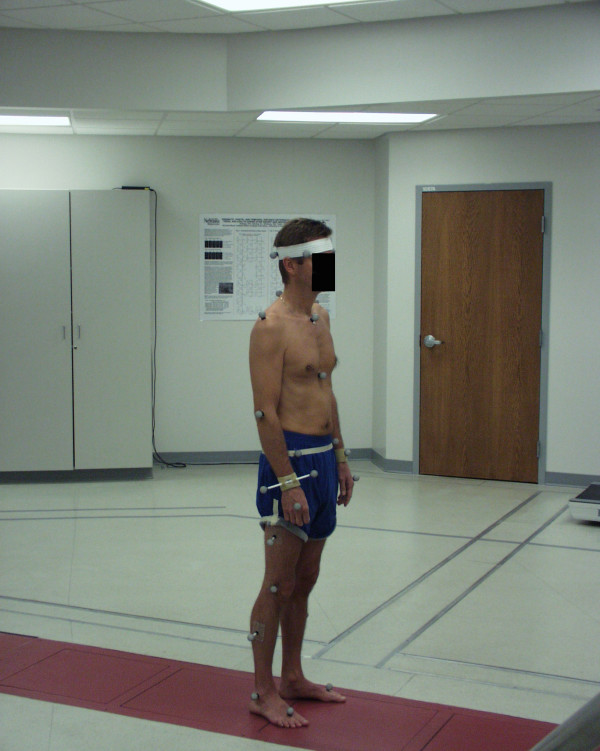
**Marker placement for computerized 3-D motion analysis**.

Marker positioning and objective measurements. Four markers, placed at the left and right temporal and occipital regions, respectively, defined a 'head' segment. Additional markers over the sternal notch, xiphoid process, and spinous processes of C7 and T10, defined a 'thorax' segment to allow calculation of orthogonal angles between the two segments. The standard Vicon marker set was used for the lower extremities with a marker on each of the anterior iliac spines, centered between the posterior superior iliac spines, lateral on the thigh and shank, lateral on the knee joint and lateral malleolus and on the dorsum of the foot over the head of the second metatarsal. Figure 1. A static trial using a knee-alignment device was used to estimate knee joint centers.

A standard lower body marker set and Plug-In-Gait modeling software was used for precise calculation of repeated angle measurements from gait. [21] The precision of angle measurements for the cervical spine using the Plug-in gait modeling software has not been determined, but is assumed to be as valid as measures for the lower body. Precision of centroid position of the markers has been demonstrated to be accurate to within a millimeter (Vicon, Oxford, England).

During data collection, subjects were asked to move the head along three planes of the neck (flexion-extension, left-right rotation, left-right lateral flexion) to the point of maximum ability or tolerance. Angles between the thorax and head segments were calculated using the Plug-In-Gait full body model, and the maximum angle for each of three trials was identified for each direction of movement. The average of the three trials was used as outcome measure for maximum active range of motion in each direction.

Prior to the measurements of cervical mobility, subjects performed 10 to 15 walking trials at their self selected usual velocity. Walking speed was calculated for each trial, and the three trials closest to the subject's average walking speed were selected for analysis of the temporal distance parameters. Outcome measures included average walking speed, cadence, and bilateral step lengths.

Pain assessment. Participants rated their overall pain before and after each evaluation session, on a linear visual analog scale (VAS) with 0 representing no pain and 10 representing the most severe pain the subject had ever felt. Using the same scale, participants also rated their pain in relation to a typical day during the previous week.

### Statistical analysis

Analysis of data was performed using Student's paired t-test. Statistical significance was set at *p *< 0.05. Intraclass correlation coefficient (ICC) was used to assess intra-session reliability for each of the six cervical spine motion measures taken during both pre and post sessions. [22] The data were compared using ICC (2,1) where time was modeled as a random effect since we were interested in the reliability between any repeated measurements measured not on the same time per session.

## Results

Excellent reliability of the cervical spine measures were observed with ICC values consistently above 0.9 as detailed in Table 3.

**Table 3:** Cervical Spine Measure ICC Values

	ICC Value	
	
C-Spine Motion Variables	Pre-Session Measure	Post-Session Measure
Extension	0.979	0.987

Flexion	0.912	0.956

Left Lateral Flexion	0.983	0.963

Right Lateral Flexion	0.952	0.972

Right Rotation	0.973	0.986

Left Rotation	0.971	0.986

The analysis of data confirmed statistically significant (p < 0.005) improvement in cervical range of motion in all six planes following treatment, with the greatest average improvements in flexion-extension (54%), followed by rotation (53.5%). Table 4.

**Table 4:** Maximum Active Neck Range of Motion (degrees)

	*Pre-op*	*Post-op*	*Mean change*		*Paired t-test*	
	
	*Mean ± SD*	*Mean ± SD*	*Degrees*	*Percent*	*t statistic*	*p-value*
**Flexion**	25.2 ± 11.9	39.6 ± 12.9	14.4	57	-3.61	0.002

**Extension**	29.3 ± 13.8	44.4 ± 20.2	15.1	52	-4.16	0.0008

**L Rotation**	36.1 ± 21.0	54.1 ± 18.2	18.0	50	-4.21	0.0007

**R Rotation**	37.3 ± 16.3	59.1 ± 16.1	21.8	58	-5.78	0.00006

**L Lat Flexion**	19.4 ± 14.1	25.9 ± 16.2	6.5	34	-3.07	0.005

**R Lat Flexion**	22.9 ± 1201	32.7 ± 10.2	9.8	42	-4.97	0.0002

At follow-up, walking speed had increased by an average of 13.9 centimeters/second, with a 5.2 centimeter average increase in step length. Table 5.

**Table 5:** Temporal-Distance Gait Parameters

	*Pre-op*	*Post-op*	*Mean Difference*		*Paired t-test*	
	
	***Mean ***± ***SD***	***Mean ***± ***SD***	*Degrees*	*Percent*	*t statistic*	*p-value*
**Walking speed (cm/sec)**	98.5 ± 29.1	112.4 ± 17.4	13.9	14	-2.94	0.007

**Cadence (steps/min)**	105.9 ± 13.8	112.1 ± 7.6	6.2	6	-2.32	0.02

**Step length (cm)**	54.5 ± 11.1	59.7 ± 7.9	5.2	10	-2.79	0.009

All patients gave postoperative neck pain ratings that were significantly lower than before surgery, both for daily pain, and for how much their pain increased during exertion. Table 6.

**Table 6:** Pain Ratings (Visual-Analog Scale 0–10)

	*Pre-op*	*Post-op*	*Mean change*		*Paired t-test*	
	
	*Mean ± SD*	*Mean ± SD*	*VAS*	*Percent*	*t statistic*	*p-value*
**Typical day average**	6.2 ± 2.0	2.5 ± 1.8	3.7	-60	3.75	0.002

**Increase during test**	1.6 ± 2.4	0 ± 1.9	1.6	-100	1.82	0.05

No major complications related to treatment were documented among the participants during surgery or the postoperative period.

## Discussion

Significant improvement in three gait parameters were documented after treatment for neck pain from whiplash, a condition that because of a purported lack of diagnostic laboratory findings has been described by some authors as a social or emotional disorder in need of no treatment. [23-25]

Pain-related neck stiffness is a cardinal component of the chronic whiplash syndrome, but reliable assessment of cervical range-of-motion is highly dependent on the subject's voluntary effort. Inclinometer- or observation based techniques, or even computer-guided three-dimensional measurement systems are therefore not ideal tools to objectively confirm or monitor chronic whiplash.[26] In contrast, gait is a complex but highly automated function and therefore better suited for standardized analysis.

A clinically validated marker system [27,28] was adopted for the purpose of this investigation, and the consistency of cervical range-of-motion was confirmed through repeated measurements in each participant since kinematic reproducibility has been established as a method to differentiate healthy subjects simulating neck pain from patients with true whiplash injuries.[7,12,29] With these precautions, we consider the present findings reliable and valid.

Various kinematic abnormalities have been reported in chronic whiplash syndrome, often without conclusive evidence of their underlying cause(s). Thus, even though imaging evidence of abnormal cervical [30] or craniocervical [31] motion patterns have lead to recommendations to fuse the cranio-cervical joint complex, [32,33] it has not been shown that a causative relation exists between such radiological findings and the clinical whiplash syndrome. Other investigators have interpreted patterns of oculomotor dysfunction in whiplash patients as evidence of brainstem injury, or "disorganized neck proprioceptive activity" leading to distortion of the posture control system. [34-37] While none of the participants in this investigation had undergone specific diagnostic studies to assess brain stem function or cervical stability, the significant improvements in pain, cervical range-of-motion, and temporal-distance gait parameters illustrate that soft tissue surgery may alleviate considerable symptoms after whiplash in carefully selected patients. The findings also allow the following conclusions: (1) Upper segment pain, e.g. in chronic whiplash syndrome, may be expressed as gait and posture abnormalities;*and *(2) Computerized 3D gait analysis provides objective data for diagnosis or outcome studies in chronic whiplash.

## Competing interests

The authors declare that they have no competing interests.

## Authors' contributions

All authors participated in design and planning of the study, and read/approved the final manuscript. Patient selection and surgical interventions were performed by NAN. Data collection was performed by SDJ, and supervised by WS and GMG. Statistical analysis by WS.
